# Genotyping Analysis by RAD-Seq Reads Is Useful to Assess the Genetic Identity and Relationships of Breeding Lines in Lavender Species Aimed at Managing Plant Variety Protection

**DOI:** 10.3390/genes12111656

**Published:** 2021-10-21

**Authors:** Francesco Scariolo, Fabio Palumbo, Alessandro Vannozzi, Gio Batta Sacilotto, Marco Gazzola, Gianni Barcaccia

**Affiliations:** 1 Department of Agronomy Food Natural Resources Animals Environment, Campus of Agripolis, University of Padova, 35020 Legnaro, Italy; francesco.scariolo@phd.unipd.it (F.S.); fabio.palumbo@unipd.it (F.P.); alessandro.vannozzi@unipd.it (A.V.); 2 Gruppo Padana Ortofloricoltura S.S., Via Olimpia 41, 31038 Treviso, Italy; giobatta@gruppopadana.com (G.B.S.); marco.gazzola@gruppopadana.com (M.G.)

**Keywords:** *Lavandula*, NGS, genotyping by RAD sequencing, flavonoids, terpenes, chloroplast DNA barcoding, ancestry reconstruction, interspecific crosses, plant breeder’s rights

## Abstract

Lavender species are widely distributed in their wild forms around the Mediterranean Basin and they are also cultivated worldwide as improved and registered clonal varieties. The economic interest of the species belonging to the *Lavandula* genus is determined by their use as ornamental plants and important source of essential oils that are destinated to the production of cosmetics, pharmaceuticals and foodstuffs. Because of the increasing number of cases of illegal commercialization of selected varieties, the protection of plant breeders’ rights has become of main relevance for the recognition of breeding companies’ royalties. With this aim, genomic tools based on molecular markers have been demonstrated to be very reliable and transferable among laboratories, and also much more informative than morphological descriptors. With the rising of the next-generation sequencing (NGS) technologies, several genotyping-by-sequencing approaches are now available. This study deals with a deep characterization of 15 varietal clones, belonging to two distinct *Lavandula* species, by means of restriction-site associated DNA sequencing (RAD-Seq). We demonstrated that this technology screens single nucleotide variants that enable to assess the genetic identity of individual accessions, to reconstruct genetic relationships among related breeding lines, to group them into genetically distinguishable main subclusters, and to assign their molecular lineages to distinct ancestors. Moreover, a number of polymorphic sites were identified within genes putatively involved in biosynthetic pathways related to both tissue pigmentation and terpene production, useful for breeding and/or protecting newly registered varieties. Overall, the results highlighted the presence of pure ancestries and interspecific hybrids for the analyzed *Lavandula* species, and demonstrated that RAD-Seq analysis is very informative and highly reliable for characterizing *Lavandula* clones and managing plant variety protection.

## 1. Introduction

Lavender species *Lavandula stoechas* L. and *Lavandula pedunculata* (Mill.) Cav., belonging to the Lamiaceae family, include diploid plants (both 2*n* = 2*x* = 30 [1]). The wild forms of these species are widely distributed on the coast of countries around the Mediterranean Sea and are also cultivated worldwide using registered clonal varieties. The reproductive strategies of *L. stoechas* and *L. pedunculata* are prevalently allogamous and characterized by entomophilous pollination, although self-compatibility and autogamous events have also been reported [2]. Similar to many others belonging to the *Lavandula* genus, these species are known for their ornamental use and for the production of essential oils (EOs) rich in linalyl acetate, the fragrance of which is greatly appreciated for several purposes (i.e., cosmetics, lotions, soaps, room fragrances and food aromas) [3]. Moreover, lavender EOs are used in pharmacology, aromatherapy, and natural medicine, given their anti-inflammatory properties [4,5,6].

Given the growing economic interest around these species, the necessity for plant breeders and breeding companies to adequately register their varieties and to protect them from plagiarism is becoming increasingly important. Currently, the European guidelines for the registration and, thus, the protection of new *Lavandula* varieties are established by the Community Plant Variety Office (CPVO) CPVO/TP-194/1-Rev protocol [7]. This technical protocol applies to all varieties of Lavandula, but it is particularly adapted to four main sections namely Lavandula (e.g., *Lavandula angustifolia*), Stoechas (e.g., *L. stoechas*), Pterostoechas (e.g., *L. pinnata*) and Intersectional (that mainly includes interspecific hybrids such as *Lavandula* × *allardii*). In order to define the distinctness, uniformity and stability (DUS) requirements that should be met for the registration, the protocol provides a list of 40 morphological descriptors, including growth habit, plant size, leaf color intensity, leaf width, leaf length, spike length and flower color. The possibility of combining the morphological descriptors with molecular and/or biochemical profiles is never mentioned. As previously demonstrated in other crops, given the limits of phenotypic characterization and morphological markers, the use of molecular markers is becoming undeniably crucial [8,9,10,11]. The use of dominant markers has been reported in several studies to be helpful in assessing the genetic distinctiveness and uniformity of species belonging to the genus *Lavandula* [12,13,14,15]. However, the low reproducibility and the difficulty in associating these markers with phenotypic traits make them unsuitable for varietal registration processes. Codominant markers are instead able to overcome these limitations, and among them, SSR and SNP markers are the most commonly used markers. For example, previous studies successfully identified SSRs [16,17] strictly associated with genomic regions involved in the synthesis of Eos [18] or single nucleotide polymorphisms (SNPs) located within genes involved in the biosynthetic pathways of the main terpenes characterizing essential oils [18]. The analysis of genotypes linked to chemotypes [19,20] would allow researchers to identify the most suitable molecular markers to be used in screening analysis for breeding selection and variety registration. The use of molecular markers is also of relevant interest for marker-assisted selection (MAS) purposes: the association among molecular markers and genomic loci involved in the biosynthesis of flavonoids and other coloring compounds would allow for the correlation of specific phenotypes and genotypes.

Although different molecular approaches have been used to assess the distinctiveness of varieties of the *Lavandula* species, this genus suffers from the lack of annotated genome assemblies in international databases. However, according to Jingrui Li et al. [21], there is one genome assembly for *L. angustifolia* that is not publicly available that would simplify the identification of mapped molecular markers suitable for the above-described purposes.

The present study is focused on the application of the Restriction Site-Associated DNA (RAD) marker sequencing technology, not only to assess the extent of genetic similarity and heterozygosity/homozygosity of a core collection of 15 accessions belonging to two species of the *Lavandula* genus, but also to identify the genomic loci suitable for marker-assisted breeding (MAB) and for registration/protection of newly bred varieties. These aspects are of major interest for breeding companies and plant breeders when developing new commercial clones destined to the market.

## 2. Materials and Methods

### 2.1. Plant Materials

Fifteen samples belonging to as many breeding lines of lavender were kindly granted by Gruppo Padana S.S. (Paese, TV, Italy). Specifically, 13 *L. stoechas* and 2 *L. pedunculata* (identified as 2603 and 2605) plants were analyzed. Genomic DNA (gDNA) was isolated from 200 mg of fresh leaf tissue using the DNeasy Plant mini kit (Qiagen, Valencia, CA, USA) following the manufacturer’s protocol with a minor modification. Specifically, lysis and protein precipitation buffers were increased by 50% to facilitate the identification and, thus, the isolation of the supernatant phase containing oils, which was shown to deeply affect the quality of the gDNA in previous tests of DNA extraction. Both the quality and quantity of the genomic DNA samples were evaluated using a NanoDrop 2000c UV-Vis spectrophotometer (Thermo Fisher Scientific Inc., Pittsburgh, PA, USA) and by agarose gel electrophoresis (1% agarose/1 × TAE gel containing 1 × SybrSafe DNA stain (Life Technologies, Carlsbad, CA, USA)).

### 2.2. Restriction-Site Associated DNA Sequencing (RAD-Seq) and Data Analysis

The 15 gDNA samples were analyzed by means of restriction-site associated DNA sequencing (RAD-Seq) technology. One microgram of gDNA per individual sample was digested using the restriction enzyme MseI following the procedure described by Stevanato et al. [22]. For library preparation, digested DNA samples were diluted at a concentration of 3 ng/µL. Indexing, library preparation, sequencing, and bioinformatic analyses were performed according to the protocol described by Stevanato et al. [22]. Raw reads obtained through an Ion S5 sequencer (Thermo Fisher Scientific Inc., Waltham, MA, USA) were trimmed according to the restriction enzyme recognition motif. After quality assessment, all artifacts and Ns-containing reads were removed. Variants were called using Stacks v2.41 software [23]. SNPs were filtered to remove those meeting the following criteria: (1) SNPs with greater than 10% missing data, (2) SNPs with a sequence depth × 4, and (3) tri- and tetraallelic SNPs.

The obtained data were used for the construction of an unweighted pair group method with arithmetic mean (UPGMA) dendrogram based on Rohlf’s genetic similarity simple matching coefficient and a principal coordinate analysis (PCoA) centroid using NTSYS software v2.21 [24]. Additionally, a Bayesian clustering algorithm implemented in STRUCTURE v.2.2 [25] was used to model the genetic structure of the lavender core collection. The number of founding groups ranged from 1 to 20, and 10 replicate simulations were conducted for each value of K based on a burn-in of 20,000 and a final run of 100,000 Markov chain Monte Carlo (MCMC) steps. STRUCTURE HARVESTER [26] was used to estimate the most likely value of K, and the estimates of membership were plotted as a histogram using an Excel spreadsheet.

### 2.3. Identification of CDS-Mapping Reads and Reads Related to Terpene and Anthocyanin Biosynthesis Pathways

Reads with no missing data in the 15 samples analyzed were used to identify those sequences most likely belonging to genomic coding sequences (CDSs). No annotated assembly is available for *Lavandula*, but Jingrui Li et al. [21] reported that an assembly was deposited in NCBI. However, a search of the accession number yields no matches, and the authors did not answer our request at the time of the submission of this article. Thus, the genomes of the two phylogenetically closest species to this genus, namely, *Sesamum indicum* (GeneBank, GCF_000512975.1) and *Salvia splendens* (GeneBank: GCA_004379255.2), were considered. While the assembly of *S. indicum* was previously annotated, all the genomic loci and the resulting proteins from *S. splendens* were “hypothetical proteins” that required an additional step of annotation prior to their usage. This step was accomplished using the KAAS platform [27], the GHOSTX aligner [28] and the KEGG database for plant organisms [29]. The RAD tags were then aligned against both the *S. indicum* and *S. splendens* CDS datasets using a local BLASTn (BLAST+ 2.11.0 package) with an E-value threshold ≤1.0 × 10^−10^ and a percentage of identity ≥80%. The newly identified CDS-mapping reads were used for the construction of a UPGMA dendrogram and PCoA centroids as described in the previous section.

For reads matching genes involved in the biosynthetic pathways of terpenes and flavonoids, multiple Geneiuos alignments (Geneious software v2021.1.1, Biomatters Ltd., Auckland, New Zealand) among the 15 samples were performed to identify nonsynonymous SNPs.

### 2.4. DNA Barcoding through Sanger Sequencing for Species Determination

To highlight interspecific cross events between *L. stoechas* and *L. pedunculata*, DNA barcoding sequencing of all samples was accomplished using three chloroplast regions, namely, the *psbA-trnH* intergenic space region, the maturase K (*matK*) and ribonuclease large subunit (*rbcL*) genes. A nuclear region, namely, the internal transcribed region (ITS), was also considered. Genomic DNA amplification of the four samples considered was performed using a Veriti 96-Well Thermal Cycler (Applied Biosystems, Foster City, CA, USA) in a total volume of 25 μL of reaction mixture including 12.5 μL of MangoMix (Bioline, London, UK) with 1 μL of DNA (50 ng/μL), 2 μL of each primer (10 mM) and sterile water to reach the final volume. The following thermal conditions were adopted: 2 min at 95 °C; 35 cycles at 95 °C for 30 s, variable annealing temperature depending on the primer pair used (Table 1) for 45 s, and 72 °C for 45 s; and a final extension at 72 °C for 10 min. The PCR products were confirmed using 2% agarose/1 × TAE gels containing 1 × SYBR Safe DNA Gel Stain (Life Technologies), purified with ExoSAP-IT PCR Product Cleanup Reagent (Thermo Fisher) and sequenced on an ABI 3730XL Genetic Analyzer (Applied Biosystems). The obtained chromatograms were then assessed using Geneious Prime software, and sequences were trimmed at the 5′ and 3′ positions to remove the low-quality section were primers attached, and resulting ITS chromatograms were analyzed with “Heterozygote Plugin” version 2.0.0 (Biomatters) add-on to identify heterotic positions and then manually checked. The resulting sequences were aligned based on the barcoding region and concatenated for each sample. The resulting multiple alignment was used for the construction of a neighbor-joining tree using the Juke–Cantor algorithm, and polymorphic sites were used to create a logo graph. Bioinformatics analyses were conducted using Geneious Prime software plug-ins.

## 3. Results

### 3.1. RAD-Seq and Genetic Similarity Analyses

A RAD-Seq analysis was performed using 15 samples obtained from an equal number of breeding lines that belong to a core collection of the *Lavandula* genus. The sequencing produced a total of 44,219,948 raw reads with an average of 2.9 million reads per sample. After quality assessment and adapter trimming, we obtained 42,610,020 reads that were used for the creation of a catalog of 622,153 consensus loci and then used for variant calling as a reference. An initial pool of 43,271 SNPs was first identified. Then, after the filtering step, in which sequences with at least one missing value in one sample were discarded, 16,228 SNPs distributed in 14,922 RAD sequence tags were retained as all of them were shared in all samples.

**Figure 1:**
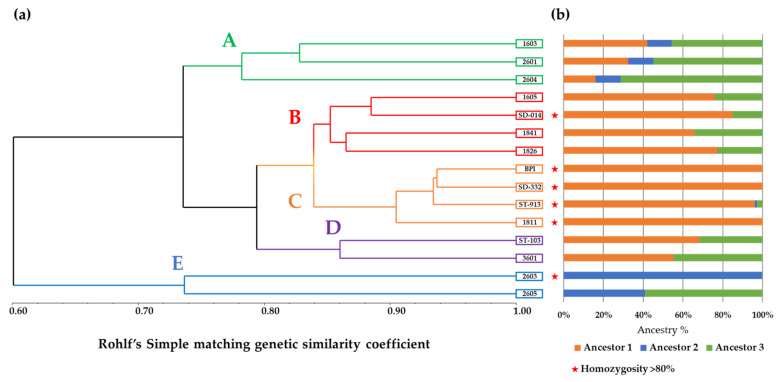
(**a**) UPGMA dendrogram based on the pair-wise genetic similarity matrix highlighting five main “Clusters” for the no missing values containing dataset. (**b**) STRUCTURE software histogram for K = 3 of 15 individuals of Lavandula with a no missing values containing dataset (“red star” symbol labels individuals with homozygosity >80%).

**Figure 2:**
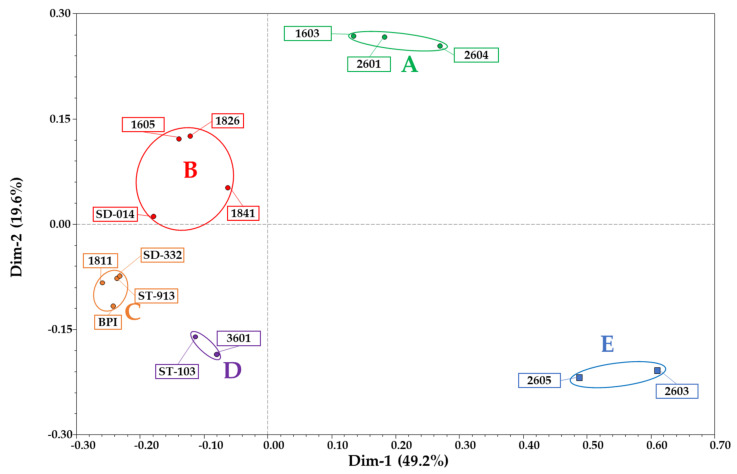
Principal Coordinate Analysis (PCoA) [24], based on the eigenvectors calculated starting from the genetic similarity matrix and highlighting the 5 mains “Clusters” (A to E) identified for the 15 analysed samples of *Lavandula*.

The analysis of the average genetic similarity (GS), which was calculated in all pairwise comparisons among the 15 sequenced samples, is reported in Table 2. Overall, GS ranged from 51.6 to 93.7% (1811 vs. 2603” and “BPI vs. SD-332”, respectively), whereas the average GS among the entire pool of samples was 74.8 ± 1.0%. The number of discriminative polymorphic sites among the most similar genotypes was 1966 SNPs, whereas that calculated among the most dissimilar was 9566 SNPs, both considering heterozygous loci. The UPGMA dendrogram grouped the 15 samples into five clusters named “Cluster A” to “Cluster E” (Figure 1), where the latter included the two *L. pedunculata* samples. From these findings, the mean genetic similarity was calculated among and between the identified groups, as reported in Table 3. The GS calculated within the clusters ranged from 73.7% in “Cluster E” to 92.0 ± 0.8% in “Cluster C”, whereas the GS among groups ranged from 56.6 ± 1.3% (“Cluster C” vs. “Cluster E”) to 83.9 ± 0.6% (“Cluster B” vs. “Cluster C”). Moreover, due to the low genetic similarity between “Cluster E” and the other four subgroups, as shown by the UPGMA dendrogram, a comparison between this cluster and the other main group of 13 samples was also made. “Cluster A + B + C + D”, which is located in one main arm of the dendrogram with a within mean genetic similarity of 79.7 ± 0.7%, exhibited an observed genetic similarity equal to 60.1 ± 1.0% when compared to “Cluster E”. Considering the number of SNPs with uncommon alleles between the *L. stoechas* and the *L. pedunculata* groups, 162 SNPs were found to have one allele in the 13 samples of “Cluster A + B + C + D” and the other allele in the two samples of “Cluster E”. The PCoA grouped samples in different spaces of the diagram with Dimensions 1 and 2 representing 49.2% and 19.6%, respectively, and overall, 68.8% of the molecular variation in total (Figure 2). From the ancestry composition reconstruction analysis, a maximum ΔK value at K = 3 was found (ΔK = 260.07, as shown in Supplementary Figure S1). Thus, an equal number of putative ancestors were hypothesized with a membership of ancestry ranging from 0 to 100%, 0 to 99.8% and from 0 to 71.3%, respectively. Notably, “Ancestor 1” had no membership in samples 2605 and 2603, for which “Ancestor 2” was greater than 40%. In contrast, “Ancestor 3” had no membership in samples BPI and ST-913 and less than 5% in samples 1811, SD-332 and 2603 (see Figure 1).

Beyond the genetic similarity estimates, the observed homozygosis (Obs. Ho) of each sample was also estimated (see Table 2). The highest homozygosity was observed in sample “BPI” (90.1%), and the lowest (60.1%) homozygosity was observed in sample “2601”. The mean homozygosity among all samples was 78.7 ± 2.2%. As for genetic similarity, homozygosity was also calculated for each of the five identified clusters (see Table 3) with values ranging from 66.4 ± 3.7% to 86.7 ± 1.9% (“Cluster A” and “Cluster C”, respectively) and a mean value for group “A + B + C + D” equal to 78.5 ± 2.4%.

### 3.2. CDS-Matching Reads Identification

16,228 RAD tags (filtered for missing data) were aligned against the exome of both *S. indicum* and *S. splendens* by means of BLASTn. Concerning the sesame exome, 2618 reads mapped on 2907 CDS, whereas, in the case of scarlet sage, a much higher number of reads (4239) aligned on 6534 CDS. Almost the totality of reads mapping on the sesame exome also mapped on the scarlet sage one (2286) (Table 4 and Supplementary Tables S1–S2).

CDS-mapping reads were then used to perform a more stringent genetic similarity analysis following the procedure previously described for the entire SNP dataset.

We created two subsets composed of those reads matching on sesame and scarlet sage exomes and we used them to calculate the pairwise genetic similarity amongst the 15 samples under study. Table 5 shows the pairwise genetic similarity matrix calculated based on sesame exome matching reads, whereas Table 6 illustrate the mean GS over clusters. Results obtained for scarlet sage are reported in Supplementary Figure S2. These estimates overall ranged from minimum values of 56.4% (sesame) and 55.5% (scarlet sage) detected comparing samples “1811” and “2603”, to maximum values of 94.2% (sesame) and 94.3% (scarlet sage) obtained from the comparison of samples “BPI” and “ST-913”. The average genetic similarity among all 15 samples was equal to 76.6 ± 0.9% for sesame-matching reads dataset and 76.2 ± 0.9% for the scarlet sage one. In general, the two genetic similarity analyses performed on datasets constituted by exome matching reads yielded highly similar results both in sample clustering and in pairwise genetic similarity percentages. The only differences observed were in the UPGMA dendrogram based on the dataset containing the reads that matched the *S. splendens* exome, in which the disposition of samples “1841” and “1826” changed from those constructed using the other two datasets (see “Cluster-Bb” in the Supplementary Figures S3–S6). Moreover, it was observed that the GS calculated within clusters was slightly higher in the matrices calculated using the exome matching read datasets than in those calculated using the no missing data containing dataset. Something similar was observed in the estimation of homozygosis, which was generally 0.5% higher in the analyses based on exome-matching reads than in those based on the whole 16,228 SNP dataset. The only exceptions were “Cluster D” and “Cluster E”, which showed homozygosity values slightly lower when considering the former dataset (see Table 5 and Table 6 and Supplementary Figure S2).

### 3.3. BLASTn Analysis for Terpene and Flavonoid Pathway-Related Gene Investigation

From the BLASTn analysis performed using the RAD tags of the 15 *Lavandula* accessions against the *S. indicum* and the *S. splendens* exomes, among the CDS-mapping reads, we selected a subgroup of sequences that aligned against genes involved in the biosynthetic pathways of terpenes and flavonoids.

In *S. indicum*, a total of nine matches were discovered for the flavonoid biosynthetic pathway and 20 for the terpene biosynthetic pathway. From the multiple alignments of the biallelic lavender reads of the 15 samples, six RAD tags presented synonymous mutations, 26 were nonsynonymous and four coded for STOP codons that were restored in three cases to a coding triplet. However, in one case, it was maintained for both alleles (RAD-tag encoded 8036 matching the 1,4-dihydroxy-2-naphthoyl-CoA synthase, accession ID: XP_011071094.1). Moreover, in *S. splendens*, 33 and 61 RAD tags matched sequences related to the flavonoid and terpene biosynthetic pathways, respectively. Similar to that performed for the matches identified in sesame pathways, multiple alignments were performed only considering the lavender RAD tags. From this investigation, 16 polymorphic sites coded for synonymous mutations, 62 were nonsynonymous and 2 coded for STOP codons. One mutation was restored in some samples to an arginine coding triplet, whereas the other maintained the missense triplet in the less frequent SNP. From the two analyses performed on the sesame and scarlet sage exomes, 7 and 17 matches were common for the flavonoid and terpene pathways, respectively. Summary statistics of the BLASTn analyses for the results of the biosynthetic pathway are reported in Table 4, BLASTN resulting matches against *S. indicum* for the biosynthetic pathways and amino acids substitutions after multiple alignments are reported in Table 7, BLASTN resulting matches against *S. splendens* for the biosynthetic pathways and amino acids substitutions after multiple alignments are reported in Supplementary Table S3, and complete BLASTN results are available in Supplementary Tables S4–S5.

### 3.4. Sanger Sequencing and DNA Barcoding Analysis

**Figure 3:**
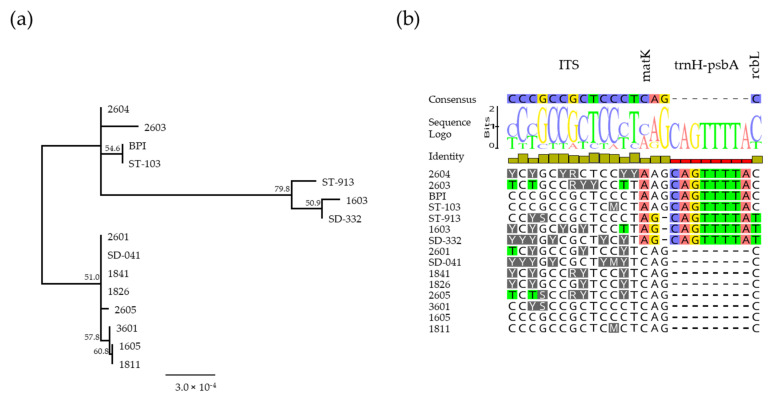
(**a**) Neighbour Joining tree based on the polymorphic sites among ITS nuclear region, and matK, trnH-psbA and rbcL chloroplast barcoding regions. Bootstrap values are reported. (**b**) LOGO representation of polymorphic sites identified among the 15 *Lavandula* accessions analysed for the DNA barcoding.

The analysis of DNA barcoding sequences commonly used in molecular taxonomy was conducted to verify the clustering reliability of the putative interspecific crosses hypothesized after ancestor membership reconstruction. The obtained sequences were 318 bp (*psbA-trnH*), 644 bp (*rbcL*), 273 bp (ITS) and 692 bp (*matK*) long, and the total concatenated sequence alignment among the four samples considered was 1926 bp long. The majority of the aligned sites were conserved, but few insertions, SNPs or heterozygous positions (ITS) were found. The different site numbers ranged from 1 (e.g., “1826” vs. “1841”) to 20 (“SD-332” vs. “2605”) among the pairwise comparisons of the aligned sequences, whereas the total number of polymorphic sites in the alignment was equal to 25. The results obtained from the neighbour-joining tree construction revealed that samples were clustered in three main subgroups, but no concordances were observed with the previously obtained results based on the RAD-Seq dataset (see Figure 3).

## 4. Discussion

### RAD-Seq-Based Genetic Similarity and Ancestral Composition Reconstruction

The use of molecular markers for genotyping analyses is currently one of the main tools in plant breeding and variety protection. Not only has this approach evolved in terms of informativeness during the late years, moving from dominant to codominant PCR-based and then to NGS-derived molecular markers, but it has also increased in the number of obtainable data and the robustness/informativeness of the resulting assays.

Indeed, RAD-Seq technology has been used for different applications in crop plant science, ranging from QTL mapping in crop species [35,36,37] to Mendelian gene mapping [38,39] and marker-assisted breeding (MAS) [9,40,41,42]. This technique has also been used for crop variety identification [43] and phylogeny [44] studies, and population structure analyses [45]. In our study, we aimed to show the potential of the RAD-Seq approach in accessing the genetic identity or similarity and distinctiveness in *Lavandula* accessions, and at identifying putative genomic loci for use in breeding schemes, registering or patenting plant varieties and novelties, and protecting plant breeders’ rights.

The great number of data points (42,610,020 total reads, 2,834,001 reads on average per sample) allowed us to investigate both the relatedness degree existing among the DNA samples and the SNP variants possibly linked to the biosynthesis of flavonoids and terpenes. To confer the robustness of the analysis, of the 43,271 SNP sites originally identified, only those with no missing data were retained (16,228). Notably, among the 27,043 RAD tags that were filtered and removed from the initial dataset, 1044 had missing values in the *L. pedunculata* samples that were instead scored among the 13 individuals of *L. stoechas*. From these findings, it could be hypothesized that these loci are likely species-specific and could, therefore, be used for species discrimination. The filtered marker dataset used for the genetic similarity analysis allowed us to group the 15 samples into five main clusters. Moreover, the GS calculated within “Cluster A + B + C + D” was comparable to that calculated within “Cluster E”, whereas the GS calculated between the two groups was lower, which is consistent with the fact that two different species were represented. Regarding the ancestral membership reconstruction, the number of K = 3 derived from the STRUCTURE software analysis was used to divide the 15 samples of the core collection of *Lavandula* into three main ancestors, showing membership percentages that were consistent with that obtained from the genetic similarity analysis. From these results, two main ancestors for accessions of *L. stoechas* were hypothesized, whereas one main ancestor mostly represented the *L. pedunculata* ancestry. The fact that admixed memberships were present among samples belonging to different species can be explained by a few factors. In the first hypothesis, interspecific crosses can be present between the two considered species, a fact that is highly probable as they are reported to be cross-fertile and belong to the taxonomic section Stoechas of the genus *Lavandula* [46,47]. Notably, *L. stoechas* and *L. pedunculata* have been reported to be phylogenetically related and very close to one another. These species are so closely related that *L. pedunculata* has been considered in the past as a subspecies of *L. stoechas* but was subsequently reassigned as a different species [46]. Then, the possibility of conserved loci among the analysed samples is possible and could relate to common ancestral genotypes between the two species. Another consideration, excluding the possible biological explanations, is that the use of a reduced and filtered dataset based exclusively on loci that are shared among all analysed samples and presenting no missing data could have resulted in a reduced capability of the molecular information in assessing the correct ancestry reconstruction. Specifically, missing data could be caused by the missed sequencing of the genomic fragment in one or more samples or by the absence of the restricted genomic region due to a polymorphic nucleotide in the restriction site. In the first case, the missing information is not usable for genomic or statistical comparisons among the samples. In the second case, however, the absence of the data is an allele itself that could be used in species determination investigation. To address this issue, the use of an assembled genome of both or at least one of the analysed species would be useful.

To confirm the first hypothesis, a barcoding analysis based on Sanger DNA sequencing of three cytoplasmic regions and one nuclear region was performed on the 15 samples of the core collection of *Lavandula*. The results obtained showed very few polymorphic sites among the analysed sequences with a maximum number of 20 among 1926 sequenced base pairs, which was approximately 1% of the total. These results were not in agreement with those obtained from the GS clustering or the ancestral reconstruction analysis performed by STRUCTURE. However, the difference can be explained by the different types of analysis performed and the nature of the molecular information used. The analysed cytoplasmic DNA regions, including both genic and intergenic sequences, are inherited by the maternal parent, so they are not suitable for phylogenetic analyses in interspecific crosses. Thus, the ITS nuclear region was also considered and found to be able to discriminate the two *L. pedunculata* individuals from the other 13 accessions of *L. stoechas* (Supplementary Figure S7). Therefore, based on the observed data, the use of a DNA barcoding strategy in determining interspecific crosses is useless or much less informative than the RAD-Seq technology.

BLASTN analysis was also performed using the 16,228 RAD tags as queries against the *S. indicum* RefSeq genome and *S. splendens* newly assembled genome to identify the RAD tags most likely attributable to gene coding sequences and possibly phenotype related. A total of 16.1% of the reads matched the CDS from sesame, whereas 26.1% of the reads matched the exome regions of scarlet sage. Based on this analysis, it was possible to filter the original RAD-Seq dataset to a limited number of sequences that were subsequently used for a new and more stringent genetic similarity analysis. The resulting data used to calculate the genetic similarities and relationships among accessions and the extent of heterozygosity/homozygosity of all accessions showed no relevant differences compared with findings from the analysis of the nonfiltered dataset, with the exception of a few cases that can be explained by a higher similarity of the conserved exonic regions. In addition, the two PCoAs derived from these reduced datasets were consistently similar to the PCoAs performed using the initial 16,228 markers (Figure 2 and Supplementary Figures S5 and S6), demonstrating once again the discriminative ability of the method used in these analyses and the relatedness of expressed and nonexpressed regions among the genomes in genotyping studies [48,49,50].

Regarding the heterozygosity estimates, it was observed that accessions showing a greater homozygosity were also those with the highest ancestral membership percentage to one or the other ancestors probably due to selfing or inbreeding reproductive strategies. The fact that few of the analysed samples exhibited high levels of heterozygosis can be explained by the presence of interspecific crosses between the two species considered in this study. Notably, those samples with greater membership percentages with one of the three identified ancestors were also those with greater homozygosity (“Cluster C” and samples “SD-014” and “2603”), whereas the admixed samples showed the highest degree of heterozygosity (“Cluster A”). Consistent with the reproduction strategy of these species, autogamy rarely occurs in natural populations [2]. However, it has been reported that these species are self-compatible, so breeding lines can be obtained by increasing homozygosity levels through controlled self-pollinations. Moreover, highly heterozygous breeding lines can be maintained at their heterozygous status and can be vegetatively reproduced by cutting, thus maintaining the phenotypic characteristics of the line and their heterotic vigour and avoiding segregation after self-pollination or recombination from cross-pollination with other lines. Moreover, the use of interspecific crosses between *L. stoechas* and *L. pedunculata* is used to transfer phenotypic traits that are desired to be maintained for commercial purposes; thus, “hybrids” are reproduced by cutting to avoid loss of desired traits, which could explain the combined results of ancestry reconstruction with homozygosity. In conclusion, the results and type of data obtained through the method proposed in this study highlighted the informativeness of the approach used and showed how genotyping-by-sequencing thorough RAD-Seq is highly informative and could be considered a useful tool to be used in combination or in place of other genotyping technologies based on PCR-based molecular markers, both dominant and codominant. Further studies are needed to confirm whether the identified SNPs are associated with phenotypic evidence.

Some findings about the STOP codons in genes involved in the synthesis of terpene precursors, including 1,4-dihydroxy-2-naphthoyl-CoA synthase, a phylloquinone precursor [51], and phosphomevalonate kinase (PMK), an inositol-diphosphate precursor [52], were particularly interesting, but further studies are needed to investigate and validate their gene function, expression, and compound synthesis to possibly correlate genotypes to chemotypes and phenotypes. This approach would be useful for MAB, including MAS approaches, and particularly for variety registration and protection.

The polymorphism information contents, and molecular profiles obtained through the technology adopted in our research project, would enable us to guarantee the breeders’ rights of the analysed varieties and to legally protect them from any theft or embezzlement and commercialization by companies competing with the rights owner’s breeders. This aim would be further improved by the creation of specific molecular assays based on prebuilt arrays able to simplify and speed-up routine screenings. Most importantly, it would be helpful to legally define the genetic similarity/diversity thresholds between commercialized varieties able to consider them distinguishable or essentially derived to avoid misunderstandings or legal issues in the genus *Lavandula*, as has already been applied or suggested for other crops [53,54,55].

## 5. Conclusions

In conclusion, genotyping analysis by RAD-Seq reads was found to be useful for assessing the genetic identity and relationships of breeding lines in lavender species aimed at managing plant variety protection. Furthermore, the described approach provides an informative characterization analysis which would help with lavender varieties registration procedures, which are now based on the only phenotypic evaluation, with no genetic investigation needed.

## Figures and Tables

**Table 1:** List of primers used for each chloroplast (cpDNA) and nuclear (nuDNA) marker with their nucleotide sequence, and reference source.

Marker	Primer Name	Primer Sequence (5′-3′) *	T*_a_* (°C)	References
*rbcL* gene (cpDNA)	rbcL_F	GCAGCATTYCGAGTAASTCCYCA	55	[30]
	rbcL_R	GAAACGYTCTCTCCAWCGCATAAA		[30]
*matK* gene (cpDNA)	matK4La	CCTTCGATACTGGGTGAAAGAT	55	[31]
	matK1932Ra	CCAGACCGGCTTACTAATGGG		[31]
*trnH-psbA* (cpDNA)	psbA3′f	GTTATGCATGAACGTAATGCTC	55	[32]
	trnHf	CGCATGGTGGATTCACAATCC		[33]
ITS1 (nuDNA)	ITS5	GGAAGTAAAAGTCGTAACAAGG	55	[34]
	ITS2	GCTGCGTTCTTCATCGATGC		[34]

* Y: C or T; S: G or C; W: A or T; T*_a_*: primers’ annealing temperature.

**Table 2:** Genetic Similarity matrix of 15 *Lavandula* individuals based on 16,228 SNPs with no missing data, and relative observed homozygosity (Obs. Ho) and heterozygosity (Obs. He).

	Obs. Ho	Obs. He		Sample			Genetic Similarity (GS)												
1603	66.1%	33.9%	Cluster A	1603	100.0%														
2601	60.1%	39.9%		2601	82.8%	100.0%													
2604	72.8%	27.2%		2604	78.9%	77.6%	100.0%												
1605	76.4%	23.6%	Cluster B	1605	79.8%	77.8%	73.8%	100.0%											
1841	78.8%	21.2%		1841	77.9%	75.7%	71.1%	86.4%	100.0%										
1826	77.9%	22.1%		1826	79.2%	76.9%	74.1%	87.4%	86.5%	100.0%									
SD-014	85.5%	14.5%		SD-014	76.5%	74.3%	70.6%	88.5%	83.9%	83.3%	100.0%								
BPI	90.1%	9.9%	Cluster C	BPI	74.6%	72.3%	68.2%	82.8%	79.1%	83.7%	83.8%	100.0%							
ST-913	84.8%	15.2%		ST-913	75.0%	74.2%	70.4%	85.7%	81.1%	86.5%	83.5%	93.3%	100.0%						
SD-332	82.4%	17.6%		SD-332	75.9%	74.4%	70.5%	83.5%	79.1%	84.8%	85.4%	93.7%	93.5%	100.0%					
1811	89.7%	10.3%		1811	75.1%	72.1%	67.7%	86.0%	85.6%	85.9%	86.4%	89.7%	92.2%	89.5%	100.0%				
ST-103	77.6%	22.4%	Cluster D	ST-103	75.7%	72.9%	69.4%	80.7%	79.4%	76.9%	82.8%	83.2%	82.2%	82.3%	82.0%	100.0%			
3601	78.1%	21.9%		3601	72.2%	70.4%	67.8%	78.0%	76.5%	75.2%	80.0%	76.3%	77.2%	77.2%	80.9%	86.0%	100.0%		
2603	87.8%	12.2%	Cluster E	2603	63.0%	64.0%	65.9%	55.4%	58.8%	56.2%	54.0%	53.9%	53.9%	53.4%	51.6%	54.9%	53.1%	100.0%	
2605	71.6%	28.4%		2605	67.6%	68.9%	69.3%	62.0%	66.4%	62.9%	60.0%	58.9%	61.0%	60.3%	59.7%	64.0%	63.8%	73.7%	100.0%
					1603	2601	2604	1605	1841	1826	SD-014	BPI	ST-913	SD-332	1811	ST-103	3601	2603	2605
					Cluster A			Cluster B				Cluster C				Cluster D		Cluster E	

**Table 3:** Average genetic similarity of clusters identified through the construction of the UPGMA dendrogram, and average observed homozygosity (Avg. Obs. Ho).

Avg. Obs. Ho	Cluster	Avg. Genetic Similarity (GS)					
66.4% ± 3.7%	Cluster A	79.8% ± 1.6%					
79.7% ± 2.0%	Cluster B	75.6% ± 0.9%	86.0% ± 0.8%				
86.7% ± 1.9%	Cluster C	72.5% ± 0.8%	83.9% ± 0.6%	92.0% ± 0.8%			
77.9% ± 0.2%	Cluster D	71.4% ± 1.1%	78.7% ± 0.9%	80.2% ± 1.0%	86.0% ± N/A		
79.7% ± 8.1%	Cluster E	66.4% ± 1.1%	59.4% ± 1.5%	56.6% ± 1.3%	58.9% ± 2.9%	73.7% ± N/A	
78.5% ± 2.4%	A + B + C + D					60.1% ± 1.0%	79.7% ± 0.7%
		Cluster A	Cluster B	Cluster C	Cluster D	Cluster E	A + B + C + D

**Table 4:** Summary statistics of the BLASTN analysis of the RAD-Seq reads against the exomes of *S. indicum* and *S. splendens*. Statistics information of the flavonoids and terpenes pathways involved genes is also reported.

BLASTn Result	RAD-Tags (*n*)	CDS (*n*)	Protein Products (*n*)	Avg. Identity (%)	Avg. Length (bp)	Avg. E-Value	Avg. Bitscore	Avg. Score	Avg. Mismatches (*n*)	Avg. Identity (*n*)	Avg. Positive Positions
Exome *S.ind*	2618	2907	2077	87.3	64.4	5.33 × 10^−12^	80.2	87.5	8.2	56.2	87.3
Flavonoids	15	14	10	86.7	67.1	1.04 × 10^−12^	82.1	89.6	8.9	58.2	86.7
Terpenes	20	24	19	86.0	62.9	6.20 × 10^−12^	74.3	81.0	9.0	53.9	86.0
Exome *S.sp*	4239	6534	1215	88.7	64.2	2.90 × 10^−12^	83.8	91.5	7.3	56.9	88.7
Flavonoids	33	40	18	87.4	66.0	2.41 × 10^−12^	82.5	90.1	8.3	57.6	87.4
Terpenes	61	65	28	88.9	65.6	1.45 × 10^−12^	86.6	94.7	7.3	58.3	88.9

**Table 5:** Genetic Similarity matrix of 15 Lavandula individuals based the BLASTN analysis against *S. indicum* exome, and relative observed homozygosity (Obs. Ho) and heterozygosity (Obs. He).

Obs. Ho	Obs. He					Genetic Similarity (GS)												
68.3%	31.7%	Cluster A	1603	100.0%														
60.4%	39.6%		2601	83.1%	100.0%													
73.7%	26.3%		2604	79.6%	77.3%	100.0%												
78.3%	21.7%	Cluster B	1605	81.2%	78.5%	74.8%	100.0%											
86.1%	13.9%		SD-014	77.9%	75.5%	72.3%	88.9%	100.0%										
78.2%	21.8%		1841	79.7%	77.5%	73.1%	86.9%	85.1%	100.0%									
79.5%	20.5%		1826	81.0%	78.3%	75.9%	87.0%	84.5%	88.1%	100.0%								
90.4%	9.6%	Cluster C	BPI	76.4%	74.0%	70.6%	83.5%	85.8%	80.6%	84.3%	100.0%							
85.3%	14.7%		ST-913	76.9%	76.1%	72.7%	86.2%	85.5%	82.0%	87.4%	94.2%	100.0%						
83.2%	16.8%		SD-332	77.3%	76.3%	72.9%	83.9%	86.9%	80.4%	85.1%	93.9%	93.7%	100.0%					
89.7%	10.3%		1811	77.5%	74.3%	70.4%	86.8%	87.9%	86.2%	86.6%	90.4%	92.6%	90.0%	100.0%				
78.5%	21.5%	Cluster D	ST-103	77.5%	74.7%	71.3%	82.5%	84.2%	81.6%	79.0%	84.0%	83.8%	83.3%	84.1%	100.0%			
77.3%	22.7%		3601	75.0%	73.2%	70.4%	79.4%	80.9%	78.5%	76.6%	77.8%	78.9%	78.4%	82.2%	87.0%	100.0%		
87.0%	13.0%	Cluster E	2603	65.8%	67.4%	68.9%	59.0%	58.9%	63.0%	61.1%	57.9%	58.2%	58.1%	56.4%	58.7%	57.8%	100.0%	
70.2%	29.8%		2605	69.1%	70.4%	70.9%	64.1%	63.5%	69.2%	65.9%	61.5%	63.6%	63.3%	62.9%	66.5%	67.4%	74.3%	100.0%
				1603	2601	2604	1605	SD-014	1841	1826	BPI	ST-913	SD-332	1811	ST-103	3601	2603	2605
				Cluster A			Cluster B				Cluster C				Cluster D		Cluster E	

**Table 6:** Average genetic similarity of clusters identified through the construction of the UPGMA dendrogram, and average observed homozygosity (Avg. Obs. Ho) The standard error is also reported.

Avg. Obs. Ho	Sample	Avg. Genetic Similarity (GS)					
67.4% ± 3.9%	Cluster A	80.0% ± 1.7%					
80.5% ± 1.9%	Cluster B	77.1% ± 0.8%	86.7% ± 0.7%				
87.2% ± 1.7%	Cluster C	74.6% ± 0.7%	84.9% ± 0.6%	92.5% ± 0.8%			
77.9% ± 0.4%	Cluster D	73.7% ± 1.1%	80.3% ± 0.9%	81.6% ± 1.0%	87.0% ± N/A		
78.6% ± 8.4%	Cluster E	68.8% ± 0.8%	63.1% ± 1.2%	60.2% ± 1.0%	62.6% ± 2.5%	74.3% ± N/A	
79.1% ± 2.3%	A + B + C + D					63.4% ± 0.9%	81.0% ± 0.7%
		Cluster A	Cluster B	Cluster C	Cluster D	Cluster E	A + B + C + D

**Table 7:** Multiple alignments results reporting read ID, *S. indicum* (GCF_000512975.1) accession number on NCBI database, Flavonoid/Terpenes product, KEGG ID, amino acid substitution based on the polymorphic SNP in the 15 individuals of *Lavandula*.

		FLAVONOIDS		
**Read ID**	***S. ind* CDS ID**	**product**	**KO-IDs from KEGG**	**SNP to AA Subs.**
3043	XP_011100449.1	anthocyanidin 3-O-glucosyltransferase 2	K12930	Ile -> Met
	XP_011100453.1	anthocyanidin 3-O-glucosyltransferase 2-like		
6706	XP_011090466.1	aspartate aminotransferase and glu/asp-prephenate aminotransferase	K15849	Val -> Ala
7480	XP_011089364.1	arogenate dehydratase/prephenate dehydratase 2, chloroplastic	K05359	Glu -> Val
	XP_011089363.1			
7969	XP_011094662.1	phenylalanine ammonia-lyase	K10775	Gln -> Arg
9011	XP_011089239.2	LOW QUALITY PROTEIN: 4-coumarate--CoA ligase-like 7	K01904	Gln -> Gln
9012				Gln -> Arg
9955	XP_020554052.1	putative anthocyanidin reductase isoform X2	K08695	Uncertain
	XP_011095308.1			X -> Leu
10947	XP_011069886.1	anthocyanidin 3-O-glucosyltransferase-like	K12930	Arg -> Pro
11587	XP_011077338.1	phenylalanine ammonia-lyase	K10775	His -> Tyr
		**TERPENES**		
**Read ID**	***S. ind* CDS_ID**	**product**	**KO-IDs from KEGG**	**SNP to AA Subs.**
8036	XP_011071094.1	1,4-dihydroxy-2-naphthoyl-CoA synthase, peroxisomal	K01661	X -> X
14576	XP_011096130.1	α-farnesene synthase	K14173	Gly -> Glu
6208	XP_011093795.1	β-amyrin synthase	K15813	Lys -> Glu
8386	XP_011093795.1			X -> Arg
6208	XP_011085901.1	β-amyrin synthase-like	K15813	Lys -> Glu
8386	XP_011085901.1			X -> Arg
6276	XP_011095756.1	ent-kaur-16-ene synthase, chloroplastic	N/A	Pro -> Ala
7199	XP_011083784.1	ent-kaurene oxidase, chloroplastic-like	K04122	Val -> Met
3576	XP_020550121.1	geranylgeranyl transferase type-2 subunit α 1	K09833	Leu -> Ser
10802	XP_011092247.1	gibberellin 20-oxidase-like protein	K05282	Gln -> Gln
11279	XP_011096560.1	gibberellin 2-β-dioxygenase	K04125	Phe -> Leu
10014	XP_011098626.1	gibberellin-regulated protein 4-like	N/A	Arg -> Gln
	XP_011071640.1			
4578	XP_011084658.1	isopentenyl-diphosphate Delta-isomerase I	K01823	Uncertain
6515				Phe -> Leu
13525	XP_011077171.1			Pro -> Pro
9817	XP_011075409.1	probable NAD(P)H dehydrogenase subunit CRR3, chloroplastic	N/A	Trp -> Leu
14513	XP_011082816.1	probable solanesyl-diphosphate synthase 3, chloroplastic	K05356	Leu -> Phe
14513	XP_011098150.1	probable solanesyl-diphosphate synthase 3, chloroplastic isoform X2		Leu -> Phe
5640	XP_020551000.1	protein prenyltransferase α subunit, isoform X6	K14137	Pro -> Gln
	XP_020551002.1			
3603	XP_011078470.1	squalene monooxygenase	K00511	Asn -> Thr
9296	XP_011092466.1	squalene monooxygenase-like		Asp -> His
5280	XP_011092839.1	squalene synthase	K00801	Pro -> Ser
	XP_011092841.1			
4990	XP_011082248.1	vetispiradiene synthase 3 isoform X2	K14182	Asp -> Glu
14152	XP_020548233.1	isochorismate synthase, chloroplastic-like	K01851	Arg -> Met
14154				Gln -> Pro
14685	XP_020548234.1	isochorismate synthase, chloroplastic-like	K01851	Val -> Leu
14687				Thr -> Thr
15015				Lys -> Lys

## Data Availability

The data presented in this study are available within the article or as Supplementary Material and the initial dataset is available upon request.

## Supplementary Materials

The following are available online at https://www.mdpi.com/article/10.3390/genes12111656/s1, Figure S1: STRUCTURE Harvester software resulting ΔK chart; Figure S2: (a) Genetic Similarity matrix of 15 Lavandula individuals based the BLASTN analysis against *S. splendens* exome, and relative observed homozygosity (Obs. Ho) and heterozygosity (Obs. He). (b) Average genetic similarity of Clusters identified through the construction of the UPGMA dendrogram, and average observed homozygosity (Avg. Obs. Ho); Figure S3: UPGMA dendrogram of the genetic similarity calculated on the *Lavandula* reads matching the *S. indicum* exome; Figure S4: UPGMA dendrogram of the genetic similarity calculated on the *Lavandula* reads matching the *S. splendens* exome; Figure S5: Principal Coordinate Analysis (PCoA) of the genetic similarity calculated on the *Lavandula* reads matching the *S. indicum* exome; Figure S6: Principal Coordinate Analysis (PCoA) of the genetic similarity calculated on the *Lavandula* reads matching the *S. splendens* exome; Figure S7: Neighbour Joining tree based on Geneious software plug-in “multiple alignment” of ITS nuclear region of the 15 *Lavandula* individuals; Table S1: BLASTN result of the RAD-seq obtained reads of *Lavandula* against the *S. indicum* exome; Table S2: BLASTN result of the RAD-seq obtained reads of *Lavandula* against the *S. splendens* exome; Table S3: Multiple alignments results reporting read ID, *S. splendens* (GCA_004379255.2) accession number on NCBI database, Flavonoid/Terpenes product, KEGG ID assigned by KASS, amino acid substitution based on the polymorphic SNP in the 15 individuals of *Lavandula;* Table S4: BLASTN results for *Lavandula* reads matching genes involved in the Flavonoids and Terpenes biosynthetic pathways of *S. indicum*; Table S5: BLASTN results for *Lavandula* reads matching genes involved in the Flavonoids and Terpenes biosynthetic pathways of *S. splendens*.
