# Supplementary material for: Genotyping Analysis by RAD-Seq Reads Is Useful to Assess the Genetic Identity and Relationships of Breeding Lines in Lavender Species Aimed at Managing Plant Variety Protection
Source: Genes (Basel). 2021 Oct 21;12(11):1656. doi: 10.3390/genes12111656 (PMC8621978; doi:10.3390/genes12111656)
Supplement: Supplementary file 1 [file genes-12-01656-s001.zip › Supplementary Figure S2.pdf]

Supplementary Figure S2: (a) Genetic Similarity matrix of 15 Lavandula individuals based the BLASTN analysis against S. splendens exome, and relative observed homozygosity (Obs. Ho) and heterozygosity (Obs. He). (b) Average genetic similarity of Clusters identified through the construction of the UPGMA dendro-gram, and average observed homozygosity (Avg. Obs. Ho)

(a)

Obs. He			Genetic similarity (GS)														
32.3%	Cluster A	1603	100.0%														
39.3%		2601	82.5%	100.0%													
25.4%		2604	79.1%	77.0%	100.0%												
22.2%	Cluster B	1605	81.0%	78.5%	74.7%	100.0%											
21.6%		1841	79.3%	77.3%	72.5%	86.8%	100.0%										
21.4%		1826	80.7%	78.0%	75.6%	87.1%	87.4%	100.0%									
13.9%		SD-014	77.6%	75.0%	71.7%	88.6%	84.5%	83.7%	100.0%								
10.2%	Cluster C	BPI	75.9%	74.0%	69.8%	83.8%	80.5%	84.4%	85.3%	100.0%							
14.8%		ST-913	76.2%	75.4%	71.6%	86.4%	81.8%	87.0%	84.8%	94.1%	100.0%						
16.4%		SD-332	77.0%	75.8%	71.9%	84.3%	80.3%	85.3%	86.4%	94.3%	93.9%	100.0%					
10.8%		1811	76.7%	74.0%	69.5%	86.5%	85.8%	86.0%	87.2%	90.4%	92.4%	90.0%	100.0%				
21.8%	Cluster D	ST-103	77.0%	74.2%	70.8%	82.1%	81.0%	78.7%	83.4%	83.7%	83.1%	83.1%	83.3%	100.0%			
23.0%		3601	74.3%	72.4%	69.7%	79.4%	78.1%	76.2%	80.8%	77.8%	78.5%	78.5%	81.5%	87.2%	100.0%		
13.1%	Cluster E	2603	66.1%	66.9%	68.2%	58.9%	62.3%	60.1%	58.1%	56.8%	57.1%	57.0%	55.5%	58.2%	57.3%	100.0%	
28.9%		2605	68.5%	69.8%	70.2%	64.1%	68.4%	65.1%	62.5%	60.7%	62.4%	62.1%	61.7%	65.8%	66.3%	74.2%	100.0%
			1603	2601	2604	1605	SD-014	1841	1826	BPI	ST-913	SD-332	1811	ST-103	3601	2603	2605
			Cluster A			Cluster B				Cluster C				Cluster D		Cluster E	

(b)

Obs. Ho	Sample	Avg. Genetic similarity					
67.7% ± 4.0%	**Cluster A**	79.5% ± 1.6%					
80.2% ± 2.0%	**Cluster B**	76.8% ± 0.9%	86.4% ± 0.8%				
86.9% ± 1.5%	**Cluster C**	74.0% ± 0.8%	84.7% ± 0.5%	92.5% ± 0.8%			
77.6% ± 0.4%	**Cluster D**	73.1% ± 1.1%	80.0% ± 0.8%	81.2% ± 0.9%	87.2% ± N/A		
79.0% ± 7.9%	**Cluster E**	68.3% ± 0.7%	62.5% ± 1.2%	59.2% ± 1.0%	61.9% ± 2.4%	74.2% ± N/A	
79.0% ± 2.3%	**A+B+C+D**					62.7% ± 0.9%	80.7% ± 0.7%
		**Cluster A**	**Cluster B**	**Cluster C**	**Cluster D**	**Cluster E**	**A+B+C+D**
