# Supplementary material for: Genotyping Analysis by RAD-Seq Reads Is Useful to Assess the Genetic Identity and Relationships of Breeding Lines in Lavender Species Aimed at Managing Plant Variety Protection
Source: Genes (Basel). 2021 Oct 21;12(11):1656. doi: 10.3390/genes12111656 (PMC8621978; doi:10.3390/genes12111656)

Supplementary Figure S4: UPGMA dendrogram of the genetic similarity calculated on the *Lavandula* reads matching the *S. splendens* exome

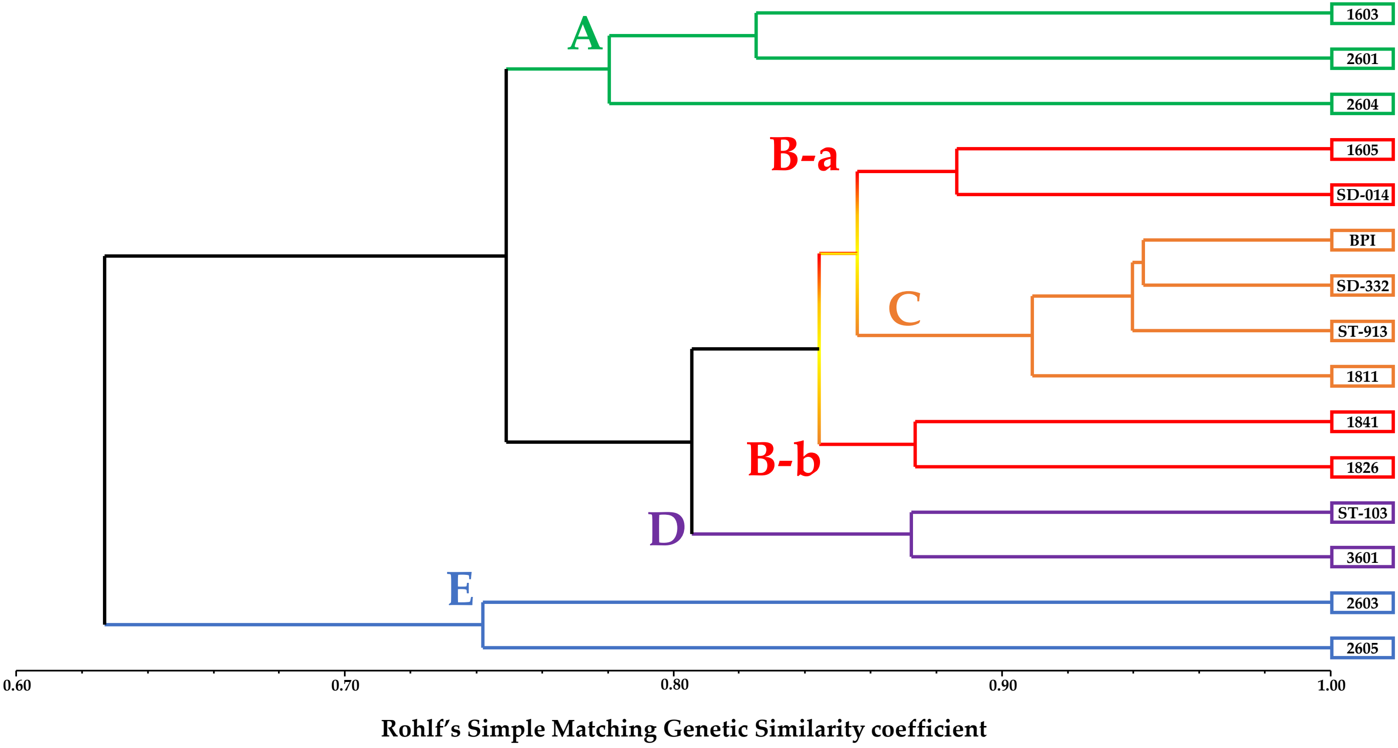

Supplement: Supplementary file 1 [file genes-12-01656-s001.zip › Supplementary Figure S4.pdf]
